# Regional Brain Volume, Brain Reserve and MMSE Performance in Healthy Aging From the NEUROAGE Cohort: Contributions of Sex, Education, and Depression Symptoms

**DOI:** 10.3389/fnagi.2021.711301

**Published:** 2021-11-12

**Authors:** Eva Pettemeridou, Eleni Kallousia, Fofi Constantinidou

**Affiliations:** ^1^Center for Applied Neuroscience, University of Cyprus, Nicosia, Cyprus; ^2^KIOS Innovation and Research Center of Excellence, University of Cyprus, Nicosia, Cyprus; ^3^Department of Psychology, University of Cyprus, Nicosia, Cyprus

**Keywords:** cognition, neuroimaging, gray matter, elderly, gender, dementia

## Abstract

**Objective**: The aim of this study was twofold. First, to investigate the relationship between age, gray matter (GM), white matter (WM), and cerebrospinal fluid (CSF) volumes, brain reserve (BR), and specific regions of interest (ROIs) with global cognitive function in healthy older adults participating in a longitudinal study on aging in the island country of Cyprus. Second, to assess the contribution of important demographic and psychosocial factors on brain volume. Specifically, the effects of sex and years of education and the association between depression symptoms on brain volume were also explored in this Mediterranean cohort.

**Methods**: Eighty-seven healthy older adults (males = 37, females = 50) scoring ≥24 on the Mini-Mental State Examination (MMSE) were included, with a mean age of 72.75 years and a mean educational level of 10.48 years. The Geriatric Depression Scale was used to assess depression. T1-weighted magnetic resonance images were used to calculate global and regional volumes.

**Results**: Age was negatively correlated with GM, WM, BR, MMSE scores, and ROIs, including the hippocampus, amygdala, entorhinal cortex, prefrontal cortex, anterior cingulate gyrus, and positively with CSF. Higher MMSE scores positively correlated with GM volume. Women exhibited greater levels of depression than men. Depression was also negatively correlated with GM volume and MMSE scores. Men had greater ventricular size than women and participants with higher education had greater ventricular expansion than those with fewer years in education.

**Conclusions**: The reported structural changes provide evidence on the overlap between age-related brain changes and healthy cognitive aging and suggest that these age changes affect certain regions. Furthermore, sex, depressive symptomatology, and education are significant predictors of the aging brain. Brain reserve and higher education accommodate these changes and works against the development of clinical symptoms.

## Introduction

Age is one of the most significant risk factors for pathological decline associated with dementia, in adults over 65 years old. Both brain and subtle neuropsychological changes may occur several years prior to the clinical manifestation of dementia (Sperling et al., 2011; Tondelli et al., 2012; Seetlani et al., 2016). Changes in brain structure, such as volume reductions, could occur prior to the onset of cognitive symptoms. The aim of the present study was to explore the association between brain volume measures and global cognitive performance, in a group of cognitively healthy older adults. The major premise for this study was that age would affect brain volume and that those changes would be detectible in otherwise cognitively healthy older adults before changes in global cognition. The present study is part of our systematic research program on the Neurocognitive Study for the Aging (NEUROAGE). NEUROAGE is the first longitudinal study on cognitive aging in Cyprus. It explores modifiable and unmodifiable factors that contribute to cognitive health in the Greek-Cypriot population of Cyprus with its unique social-cultural, linguistic and genetic characteristics (Constantinidou et al., 2012; Giogkaraki et al., 2013; Demetriou and Constantinidou, 2018; Philippou et al., 2018). The present study contributes to the existing body of literature on determining the contributions of modifiable (education, depression) and unmodifiable (sex, age) risk factors on brain volume and brain health in this cognitively healthy and genetically homogeneous Mediterranean cohort with its unique social and geopolitical characteristics (Chadjikyprianou et al., 2021).

Global measures of brain volume in aging have revealed lower volume in both gray and white matter (GM, WM, respectively), with GM volume presenting a decrease earlier than WM volume (Raz and Rodrigue, 2006). The reduction in GM volume begins in early adulthood and continues steadily throughout the adult years, while there is a steep decline in WM volume in the early 50 s (Raz and Rodrigue, 2006). A longitudinal study observed ventricular expansion (a hallmark sign of brain atrophy) in older healthy individuals and a positive association between ventricular size and age (Raz et al., 2007).

While advancing age appears to be associated with less intracranial volume (Ramanoël et al., 2018), changes in human brain volume are not uniform, but they rather appear to be selective, with specific regions of interest (ROIs) being more susceptible to age than others. For example, GM volume loss is reportedly more specific to the frontal, parietal and temporal lobe regions (Ramanoël et al., 2018). More specifically, the prefrontal cortex is one of the most prominent regions of the brain that exhibit age-related changes throughout adult life (Terribilli et al., 2011), with the ventromedial prefrontal cortex volume demonstrating greater loss in older adulthood (Kalpouzos et al., 2012). In addition, areas of the medial temporal lobe, i.e., the hippocampus, the amygdala, and the entorhinal cortex (McDonald et al., 2012; Yao et al., 2012), along with other areas that are also known to be early markers of Alzheimer’s disease, such as the inferior temporal cortex present in less volume with advancing age (Du et al., 2001; Raz et al., 2005).

Changes in brain reserve (BR) may be also associated with aging, although research in this area is very limited (Christensen et al., 2008). BR is a biological composite, which reflects differences in biological structure measured *via* brain size, including volume-to-brain ratio (VBR; Stern, 2002). This is in contrast to cognitive reserve (CR), a psychological construct, which is measured indirectly and reflects the mind’s ability to compensate for brain pathology and delay the onset of clinical symptoms (Giogkaraki et al., 2013). In a longitudinal study, change in BR, as measured by VBR, was assessed over a period of 7 years by comparing first scan images and second scan images. Results revealed that BR is not a stable biological construct and even older people who remained normal-to-normal during the study presented with some change in BR. As anticipated, the degree of change in BR was greater for those who demonstrated normal-to-mild cognitive impairment (MCI), normal-to-Dementia, and MCI-to-Dementia cases (Carmichael et al., 2007).

Global cognitive screening tools such as the Mini-Mental State Examination (MMSE; Folstein et al., 1975) can be used to quickly identify individuals with dementia by a variety of health care professionals. The MMSE is widely used in clinical, epidemiological, and research contexts, to measure global cognitive function, which includes the brief assessment of orientation, attention, memory, language, and visual-construction abilities. Studies have revealed associations between lower MMSE scores (i.e., <24) and neuroanatomical changes (Frediani, 2010; Khachiyants and Kim, 2012), such as less volume in the hippocampus (Yavuz et al., 2007; Fjell et al., 2009; Jack, 2011), the amygdala (McDonald et al., 2012; Yao et al., 2012; Dinomais et al., 2016), and the entorhinal cortex (Basso et al., 2006; McDonald et al., 2012; Yao et al., 2012), in MCI and Alzheimer’s disease.

Changes in brain structure seem to occur prior to the onset of prominent clinical symptoms. Research indicates that individuals with MCI are more likely to present with a greater reduction in brain volume before reporting impairment on daily functioning (Jack et al., 2013). Brain changes are also evident in healthy aging, as individuals without such clinical diagnoses presented with ventricular expansion that was further correlated with lower MMSE scores (Ferrarini et al., 2008; Chou et al., 2009). Yet, little is known about the sensitivity of MMSE to brain changes in healthy older adults. In fact, it has been difficult to detect associations between both global and regional brain volume with MMSE scores of equal or above 24, i.e., in healthy individuals. Most studies using the MMSE in healthy aging have focused on either global indices, such as the CSF, or specific regions (Breteler et al., 1994; Gunning-Dixon and Raz, 2003; Scarmeas et al., 2006; Janssen et al., 2007; Raz et al., 2007; Yavuz et al., 2007; Ferrarini et al., 2008; Chou et al., 2009; Kovacevic et al., 2009; Dinomais et al., 2016). However, it is not clear how these changes correlate with performance on one of the most commonly used screening tests, the MMSE, which in turn could contribute to improved clinical monitoring.

Sex may also play a pivotal role regarding changes in brain volume in healthy aging. These changes may be attributed to hormonal decreases observed in both aging sexes (Green and Simpkins, 2000; Bielecki et al., 2016). Despite the contribution of sex in brain volume in healthy aging, research is limited. Older males exhibited greater ventricular size (Coffey et al., 1999; Armstrong et al., 2019), and less volume in frontal, parietal, and temporal areas (Armstrong et al., 2019), than females.

In addition to important demographic variables such as age and sex, depression symptoms have been associated with reduced brain volume. The prefrontal cortex exhibited the greatest decrease (Janssen et al., 2007). Depression severity, along with lower MMSE scores were associated with greater ventricular expansion in both healthy elderly and individuals with Alzheimer’s disease (Janssen et al., 2007; Chou et al., 2009; Firbank et al., 2012). Furthermore, frontal and subcortical WM atrophy appeared greater in late-life depression, possibly affecting the frontal-subcortical circuits. In addition, older adults with depression and fewer years of education exhibited lower MMSE scores (Janssen et al., 2007), highlighting the contribution of formal education in cognitive health across the lifespan.

The aim of this study was to explore the association between and the predictive value of global and regional brain volume and neurocognitive changes, as measured by the MMSE in healthy aging and to identify signs that might relate to preclinical dementia and non-normal aging. It was hypothesized that: (a) age would be negatively associated with MMSE scores and global and regional measures of brain volume; (b) GM and WM would be positively correlated with and would predict MMSE performance; whereas, CSF and VBR would be negatively associated with MMSE performance; (c) Volumes in specific ROIs such as the hippocampus, the amygdala, and the entorhinal cortex would predict MMSE performance; (d) Sex would be a significant predictor of depressive symptoms, MMSE, and brain volume; (e) Years of education would predict higher levels of brain reserve and improved MMSE scores; and (f) Finally, it was expected that depression, sex, and education would predict ROIs volume.

## Materials and Methods

### Participants

The current study recruited participants from the Neurocognitive study on Aging (NEUROAGE), a longitudinal project of neurobehavioral performance, health indices, biological markers, and quality of life measures in Greek-Cypriot participants in Cyprus. NEUROAGE began in 2009 following approval by the National Bioethics Committee (EEBK/EΠ/2008/26). The study has a rolling admission process and individuals are retested on a core battery of tests every 2 years.

Out of 115 participants who had completed a magnetic resonance imaging assessment, 87 (37 male and 50 female participants) with a mean age of 72.75 years old (*range* = 60.47–90.03; *SD* = 6.48), and a mean educational level of 10.48 years (*range* = 3.00–20.00; *SD* = 4.23) were recruited in the study (for a detailed description see Table 1). All participants met the following inclusion criteria: (a) age ≥60; (b) no clinical evidence of dementia and MMSE scores ≥24; (c) good general health, no history of stroke, traumatic brain injury, or psychiatric disorder; and (d) community dwellers residing independently at home.

**Table 1:** Means, standard deviations and ranges of age, MMSE scores, years of education, GDS scores, and brain volume indexes.

	M	SD	Min	Max
Age	72.75	6.48	60.47	90.03
Years of Education	10.49	4.23	3.00	20.00
MMSE	27.22	1.77	24.00	30.00
GDS	3.18	3.04	0.00	14.00
Total Brain Volume (cm^3^)	1,285.01	133.13	1,000.77	1,631.74
Gray Matter (cm^3^)	495.99	76.33	305.88	673.02
White Matter (cm^3^)	416.36	71.49	178.56	568.60
Cerebrospinal Fluid (cm^3^)	366.21	84.82	225.12	598.61

*GDS, Geriatric Depression Scale; MMSE, Mini-Mental State Examination*.

### Measures

#### Global Cognitive Screening

The Greek adaptation of the Mini-Mental State Examination (MMSE; Fountoulakis et al., 2000) was used both as a dependent measure and as a screening measure for inclusion. MMSE assesses the following cognitive domains: orientation, attention, memory, language, and visual-construction skills. Individuals with scores higher than 24/30 were retained in the study. This cohort presented with a wide range of years of education and based on past research conducted on this cohort (Constantinidou et al., 2012), a score of 24 was representative of participants with lower education.

#### Depression Screening

Depression was assessed using the short form of the Greek adaptation of the Geriatric Depression Scale (GDS; Fountoulakis et al., 1999). GDS is a screening tool consisting of 15 self–reports, with “yes” or “no” questions. Scores of six or more suggest mild depression; while scores over 11 suggest severe depression in Greek speaking cohorts (Fountoulakis et al., 1999).

#### MRI Protocol

Magnetic resonance images were acquired with a 3.0-T scanner (Achieva, Philips Medical Systems, Best, The Netherlands). For proton excitation and signal reception, a quadrature RF body coil and a phased array 8-channel head coil were used, respectively. An isotropic, three-dimensional (3D), T1-weighted rapid acquisition gradient echo sequence (fast field echo; repetition time = 25 ms; echo time = 1.85 ms; flip angle = 30°) was utilized to acquire the whole brain, transverse MR images with an acquisition/reconstruction voxel of 1.0 mm × 1.0 mm × 1.0 mm.

#### Volumetry

Total brain, GM, WM, and CSF volumes were calculated using the Individual Brain Atlases Statistical Parametric Mapping toolbox (IBASPM: Individual Brain Atlases using Statistical Parametric Mapping Software, RRID:SCR_007110, Alemán-Gómez, 2006) under MatLab (MATLAB, RRID:SCR_001622; version R2014a; MathWorks, Natick, MA, USA). The segmented brain tissues were acquired from MR images and were created by SPM12 (SPM, RRID:SCR_007037; version 12; Ashburner, 2010), which were then entered into the IBASPM toolbox.

#### Brain Reserve

Brain reserve was quantified *via* the Ventricle-to-Brain ratio (VBR). To calculate VBR the following equation by Bigler et al. (2004) was implemented using the three volumetric indices: GM, WM, and CSF.


$$VBR=\frac{CSF}{GM+WM}\times 100$$

#### Voxel-Based Morphometry Pre-processing and Analysis

Voxel-based morphometry included four steps. First, images were segmented into GM and WM and were spatially normalized in MNI space. Then, their deformations were estimated and the imported images were aligned together. Subsequently, the generated GM images were extracted, smoothed, and were entered into statistical analyses for detecting brain regional differences (Ashburner and Friston, 2000).

Morphological changes were assessed by the general linear model. The final form of these GM images was used for statistical analysis at *p* < 0.001. The design matrices included sex, cognitive performance (MMSE groups; group one with MMSE scoring ≥27 and group two with MMSE scoring <27); while age, depression, and MMSE scores were also entered into the SPM contrast manager for possible volumetric correlations. Depending on the hypotheses, different statistical designs were conducted e.g., multiple regression (MMSE, depression, age) and *t*-tests (MMSE groups, sex), for which different covariates of no interest were entered (e.g., age, sex, depression, education). Also, to deal with brains of different sizes, total intracranial volume was used as a standard covariate of no interest. Statistical analyses were performed for the whole brain volume, and specific regions of interest: amygdala, entorhinal cortex, frontal poles 1 and 2, and the hippocampus, which were circumscribed by anatomical automatic labeling masks provided by the SPM12 toolbox. All findings were considered significant if the voxel cluster survived the family-wise error correction threshold at *p* < 0.001 (Ashburner, 2010; Konstantinou et al., 2016).

#### Statistical Analyses

Statistical Package for the Social Sciences (SPSS; Version 25) was also used to test the hypotheses. One-tailed Pearson correlations were conducted to assess associations. Multiple linear regression was conducted to evaluate the predictive value of key demographic and brain volume variables on MMSE. Independent samples *t*-tests were conducted when group comparisons were required. Due to the exploratory nature of this study, the α-level was held at 0.05.

## Results

### Aging, Cognitive Performance, and Volumetric Changes

Correlational analyses were performed to investigate the association between MMSE scores with age and volumetric indices. Prior to examining the main hypotheses, correlations were conducted to investigate the associations between the measures of global brain volume, i.e., total brain volume (TBV), gray matter (GM), white matter (WM), cerebrospinal fluid (CSF), and brain reserve (BR) demonstrated by volume-to-brain ratio (VBR). Higher rates of VBR indicate lower levels of BR. Results revealed significant associations between the TBV and all three volumetric indices, i.e., GM, *r*_(87)_ = 0.61, *p* < 0.0001, WM, *r*_(87)_ = 0.53, *p* < 0.0001, and CSF, *r*_(87)_ = 0.56, *p* < 0.0001 (Table 2). In addition, VBR negatively correlated with GM, *r*_(87)_ = −0.37, *p* < 0.0001, and WM, *r*_(87)_ = −0.44, *p* < 0.0001; and positively with CSF, *r*_(87)_ = 0.88, *p* < 0.0001 (Table 2).

**Table 2:** Correlations between age, MMSE, education, and volumetric indexes.

	1	2	3	4	5	6	7	8
1. Age	–							
2. BV	0.01	–						
3. GM	−0.42** (−0.45)**	0.61** (0.59)*	–					
4. WM	−0.24*	0.53**	0.10	–				
5. CSF	0.58**	0.56**	−0.03	−0.12	–			
6. VBR	0.68**	0.11	−0.37**	−0.44**	0.88**	–		
7. Education (*n* = 82)^a^	0.26**	0.16	−0.08	0.05	0.29**	0.25*	–	
8. MMSE	−0.20* (−0.22)*	0.18*	0.33** (0.29)**	0.08	−0.07	−0.19*	0.13	–
9. GDS	−0.05	−0.17	−0.27**	−0.03	−0.01	0.08	−0.13	−0.20*

a = Pearson’s correlations included 82 participants excluding those who had less than five education years; *p < 0.05 (one-tailed); **p < 0.01 (one-tailed); (statistical significance for partial correlations controlling for GDS). BV, brain volume; GM, Gray matter; WM, white matter; CSF, cerebrospinal fluid; VBR, volume-to-brain ratio.

Significant negative correlations were detected between depression symptoms with GM volume, and MMSE scores (Table 2). Therefore, GDS scores were partialled-out when investigating any associations with GM and MMSE scores. As expected, GM, *r*_(84)_ = −0.45, *p* < 0.0001, and WM, *r*_(87)_ = −0.24, *p* < 0.05, inversely associated with age; whereas, age positively correlated to CSF, *r*_(87)_ = 0.58, *p* < 0.0001, and VBR, *r*_(87)_ = 0.68, *p* < 0.0001 (Table 2). Findings also showed a weak but significant negative association between aging and the MMSE scores, *r*_(84)_ = −0.22, *p* < 0.05 (Table 2).

Furthermore, it was hypothesized that MMSE scores would positively correlate with all global volume measures. Partial correlations revealed associations between MMSE performance and GM volume, *r*_(84)_ = 0.29, *p* < 0.01, and with VBR, *r*_(87)_ = −0.19, *p* < 0.05. No other volumetric indices significantly correlated with MMSE scores (see Table 2).

Finally, cognitive performance on all brain volume indices was further assessed by comparing groups with high and low MMSE scores. A median-split analysis was conducted creating a group with high MMSE (MMSE ≥27, *N* = 55) and low MMSE (MMSE ≤26, *N* = 32). Analyses revealed differences only for GM volume, *t*_(85)_ = −2.45, *p* < 0.05, Cohen’s *d* = 0.54, with participants with lower MMSE scores showing lower GM volume (*M* = 469.17 cm^3^, *SD* = 83.68), than those with higher MMSE scores (*M* = 510.01 cm^3^, *SD* = 68.08; Table 3).

**Table 3:** Gender, MMSE median, and education comparisons.

	Age	BV (cm^3^)	GM (cm^3^)	WM (cm^3^)	CSF (cm^3^)	VBR	MMSE	Education	GDS
*Gender*
Men	75.47**	1351.55	502.77	416.79	423.06	47.24	27.08	12.03**	2.41*
(*n* = 37)	(6.78)	(154.28)	(94.68)	(98.53)	(80.54)	(12.34)	(1.72)	(4.32)	(2.58)
Women	70.74**	1235.78	489.23	416.04	324.14	36.25	27.32	9.34**	3.76*
(*n* = 50)	(5.49)	(88.35)	(59.68)	(42.86)	(60.13)	(8.04)	(1.81)	(3.82)	(3.26)
*MMSE*
≥27	71.93	1304.68	510.01*	421.87	365.23	39.78	-	10.58	3.75
(*n* = 55)	(5.76)	(127.95)	(68.08)	(73.02)	(86.65)	(11.07)	-	(4.53)	(3.12)
≤26	74.16	1251.24	469.17*	406.89	367.88	42.88	-	10.31	2.86
(*n* = 32)	(6.77)	(137.08)	(83.68)	(68.87)	(82.93)	(11.93)	-	(3.73)	(2.98)
*Education*
6–11 years	71.65	1259.91	501.07	403.70	347.66*	39.27	27.10	-	3.51
(*n* = 39)	(6.14)	(110.58)	(76.41)	(75.43)	(77.29)	(11.46)	(1.70)	-	(3.33)
12^+^ years	74.02	1316.01	491.28	427.16	390.38*	43.25	27.33	-	2.81
(*n* = 43)	(6.84)	(149.16)	(79.31)	(69.50)	(88.38)	(11.49)	(1.89)	-	(2.83)

GDS, Geriatric Depression Scale, *p < 0.05, **p < 0.01.

### Aging, Cognitive Performance, and Regions of Interest

**Figure 1:**
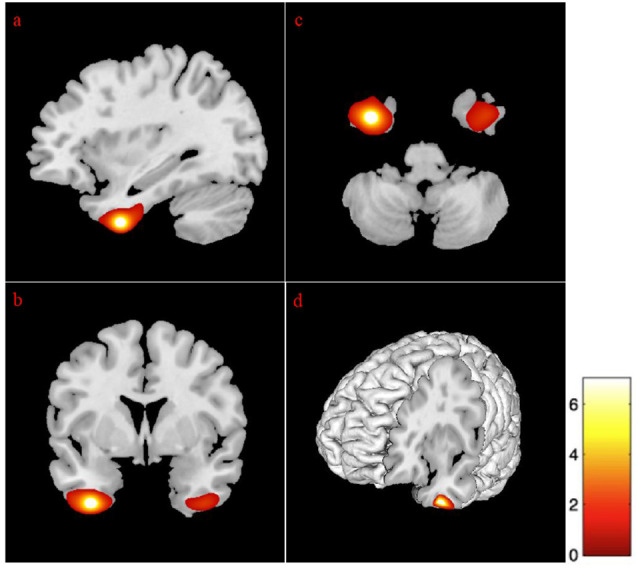
Correlation between gray matter regions and age. The position of the axes indicates a negative correlation between the left hippocampus (MNI coordinates: −18, −10, −22) and age. Color bar indicates the *t*-score for the regression slopes. **(A)** Sagittal plane, **(B)** coronal plane, **(C)** axial plane, and **(D)** 3D illustration. *p* = 0.001. MNI, Montreal Neurological Institute.

Given that the association between age and GM volume, specific regions of interest (ROIs) in the temporal and frontal regions were investigated to better characterize volumetric changes. Analyses revealed negative associations between age and the following lateral and medial temporal lobe areas: bilateral hippocampi, the right amygdala, and the anterior cingulate gyrus (see Figure 1). Also, age was negatively correlated with the right parahippocampal gyrus, as well as the left dorsal entorhinal cortex, the left temporal pole, and the right superior temporal gyrus (see Table 4).

**Table 4:** Correlations between age and ROIs.

Structure	Side	MNI coordinates			Peak- T	Age
		*x*	*y*	*z*		
Subgenual anterior cingulate cortex	L	−3	8	−8	7.91	−0.42*
Hippocampus	L	−18	−10	−22	5.89	−0.29*
Hippocampus	R	20	−14	−22	5.94	−0.29*
		18	−10	−22	5.97	−0.29*
Parahippocampal gyrus	R	21	−16	−22	5.75	−0.28*
Dorsal entorhinal cortex	L	−21	2	−16	5.88	−0.29*
Anterior cingulate gyrus	R	4	36	15	7.39	−0.39*
		4	26	26	7.16	−0.37*
		4	39	22	7.11	−0.37*
Thalamus	R	6	−15	6	7.26	−0.38*
Thalamus	L	−6	−22	4	6.67	−0.34*
Cerebellum exterior	R	45	−57	−26	6.99	−0.36*
Amygdala	R	21	3	−16	6.24	−0.31*
		21	4	−18	6.46	−0.33*
Basal forebrain	R	8	−4	−12	5.79	−0.28*
Anterior insula	L	−42	−2	6	5.79	−0.28*
Superior temporal gyrus	R	58	−24	−4	6.18	−0.30*
Temporal pole	L	−30	10	−33	5.91	−0.29*
Superior frontal gyrus medial segment	R	6	56	−2	6.49	−0.33*
Medial frontal cortex	R	6	58	−6	6.14	−0.31*
Inferior frontal gyrus (Pars orbitalis)	L	−38	26	−8	6.03	−0.30*

L, left; R, right; MNI, Montreal Neurological Institute; ROIs, regions of interest. *p < 0.001; *n* = 87.

Further correlational analyses allowed capturing volume size in other major brain regions within the frontal lobe. Specifically, the left side of both the inferior frontal lobe (pars orbitalis) and the medial frontal cortex, and the right basal forebrain and the right superior frontal gyrus (medial segment) negatively correlated with age (Table 4). Other cortical and subcortical areas that significantly correlated with age were: the left subgenual anterior cingulate cortex, the cerebellum exterior, and the thalamus (bilaterally; see Table 4).

No significant associations were evident between any of the ROIs and the MMSE. Additionally, there were no group differences between those with high and low MMSE scores on ROIs’ volumes.

### Education, Cognitive Performance, and Volumetric Changes

Education and its association with brain tissue volumes, and cognitive performance was assessed. Five participants were excluded from these analyses, due to very low educational level (less than 5 years). One-tailed correlations with 82 participants revealed that higher levels of education were moderately but significantly correlated with greater CSF volume, *r*_(82)_ = 0.29, *p* < 0.01, and VBR, *r*_(82)_ = 0.25, *p* < 0.05. Education was not associated with WM, GM, VBR, or MMSE (see Table 2). To delve further into these findings, participants were split into two groups, according to their years of education: Group 1 = 6–11 years of education (*N* = 39) and Group 2 ≥ 12 years of education (*N* = 43). Group comparisons revealed that individuals with 12+ years of education had larger ventricular size (*M* = 390.03 cm^3^, *SD* = 87.38), compared to participants with less than 12 years of education (*M* = 347.94 cm^3^, *SD* = 78.17), *t*_(80)_ = −2.34, *p* < 0.05, Cohen’s *d* = 0.51. However, no group differences were evident for GM, WM, and VBR (for means and standard deviations see Table 3).

### Sex Differences, Education, Depression, Global and Regional Volumetric Changes, and Cognitive Performance

Multivariate analyses of covariance (MANCOVAs) were performed to identify possible sex differences in brain volume and cognitive performance, controlling for age, *t*_(85)_ = 3.60, *p* < 0.01, Cohen’s *d* = 0.77 (Males, *M* = 75.47 years, *SD* = 6.78; Females, *M* = 70.74 years, *SD* = 5.49), education, *t*_(85)_ = 3.07, *p* < 0.01, Cohen’s *d* = 0.66 (Males, *M* = 12.03 years, *SD* = 4.32; Females, *M* = 9.34 years, *SD* = 3.82), and depression, *t*_(85)_ = −2.09, *p* < 0.05, Cohen’s *d* = 0.46 (Males, *M* = 2.41, *SD* = 2.58; Females, *M* = 3.76, *SD* = 3.26), differences (Table 3). There was a statistically significant difference between males and females on the combined dependent variables after controlling for age, *F*_(6,77)_ = 6.37, *p* < 0.0001, Pillai’s *T* = 0.33, partial η^2^ = 0.33, observed power = 1.00 (Table 5).

**Table 5:** Sex differences in brain volume and MMSE, controlling for age, education, and depression.

	Male	Female	Statistics					
	M (SD)	M (SD)	Pillai’s T	*df*	*F*	*P*	*η* ^2^	Observed Power
*Main Effect*
Gender			0.33	6, 77	6.37	0.0001	0.33	1.00
*Covariates*								
Age			0.48	6, 77	11.70	0.0001	0.48	1.00
Education			0.11	6, 77	1.53	0.180	0.11	0.56
Depression			0.15	6, 77	2.25	0.047	0.15	0.76
*Dependent Variables*
TBV	1,351.55 (154.28)	1,235.78 (88.35)		1, 82	17.39	0.0001	0.18	0.99
GM	502.77 (94.68)	489.23 (59.68)		1, 82	4.67	0.034	0.05	0.57
WM	416.79 (98.53)	416.04 (42.86)		1, 82	0.45	0.502	0.01	0.10
CSF	423.06 (80.54)	324.14 (60.13)		1, 82	24.42	0.0001	0.23	1.00
VBR	47.24 (12.34)	36.25 (8.04)		1, 82	12.45	0.001	0.13	0.94
MMSE	27.08 (1.72)	27.32 (1.81)		1, 82	0.54	0.464	0.01	0.11

*TBV, total brain volume*.

Sex differences were evident for TBV, *F*_(1,82)_ = 17.39, *p* < 0.0001, GM, *F*_(1,82)_ = 4.67, *p* < 0.05, and CSF, *F*_(1,82)_ = 24.42, *p* < 0.0001; with male participants exhibiting greater TBV (*M* = 1351.55 cm^3^, *SD* = 154.28), GM (*M* = 502.77 cm^3^, *SD* = 94.68), and CSF volume (*M* = 423.06 cm^3^, *SD* = 80.54), than women (TBV, *M* = 1235.78 cm^3^, *SD* = 88.35; GM, *M* = 489.23 cm^3^, *SD* = 59.68; CSF, *M* = 324.14 cm^3^, *SD* = 60.13). Additionally, males showed significantly higher VBR (*M* = 47.24, *SD* = 12.34), as compared to females (*M* = 36.25, *SD* = 8.04), *F*_(1,82)_ = 12.45, *p* < 0.01, indicating a lower level of brain reserve. Finally, there were no significant sex differences on the MMSE, *F*_(1,82)_ = 0.54, *p* = 0.464, and on WM, *F*_(1,82)_ = 0.45, *p* = 0.502 (Table 5).

**Figure 2:**
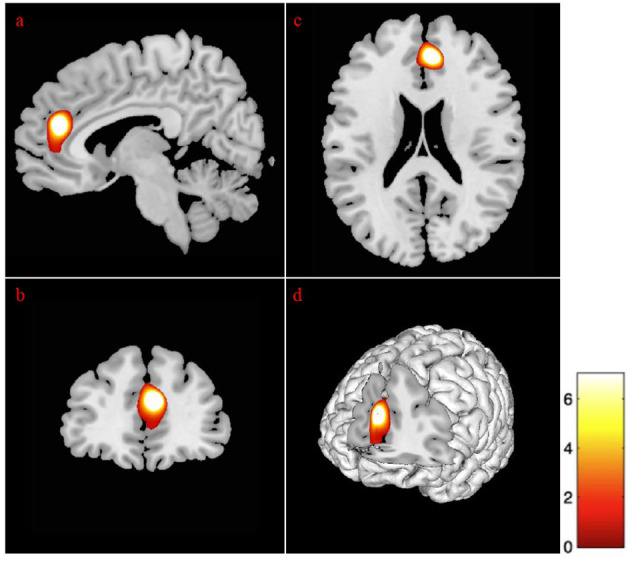
Gender differences at gray matter regions. The position of the axes indicates more atrophy of the left temporal pole (MNI coordinates: −34, 3, −44) in old women than in old men, *p* = 0.001. The position of the axes indicates more atrophy of the left inferior temporal gyrus (MNI coordinates: −36, 2, −45) in young-old women than in young-old men. **(A)** Sagittal plane, **(B)** coronal plane, **(C)** axial plane, and **(D)** 3D illustration, *p* = 0.001.

Sex differences on specific ROIs were also assessed, with females presenting with marginally less volume in the left temporal pole, as compared to males, *t*_(87)_ = 6.08, *p* < 0.01. The addition of age in the analysis for sex revealed differences at few more brain areas as depicted in Table 6. Specifically, the volumes in temporal pole, t_(87)_ = −5.94, *p* < 0.01 (Figure 2), and the inferior temporal gyrus, *t*_(87)_ = −5.96, *p* < 0.01 (Figure 2), showed less volume in young-old women compared to young-old men (young-old age *range* = 60–75).

**Table 6:** Gender comparisons.

Structure	Side	MNI coordinates			Peak- T	Older Women Vs. Older Men	Younger Women Vs. Younger Men	Women
		*x*	*y*	*z*				
Inferior temporal gyrus	L	−36	2	−45	5.96	-	5.96*	-
Temporal pole	L	−36	8	−42	5.94	-	5.94*	-
Temporal pole	L	−34	3	−44	6.08	6.08*	-	-
Temporal pole	L	−36	10	−42	5.56	-	-	5.56**

L, left; R, right; MNI, Montreal Neurological Institute; *p < 0.0001; **p = 0.001.

### Predicting Cognitive Performance

Given the above findings, stepwise regression analysis was performed to assess the predictive value of the variables that were associated with MMSE scores, i.e., age, GM, and VBR (controlling for GDS). Results showed that only higher GM volume was predictive of better MMSE performance, *B* = 0.29, *t*_(85)_ = 2.54, *p* < 0.05, *F*_(3,86)_ = 10.32, *p* < 0.05, adjusted *R*^2^ = 0.08.

### Predicting ROIs Volume

**Figure 3:**
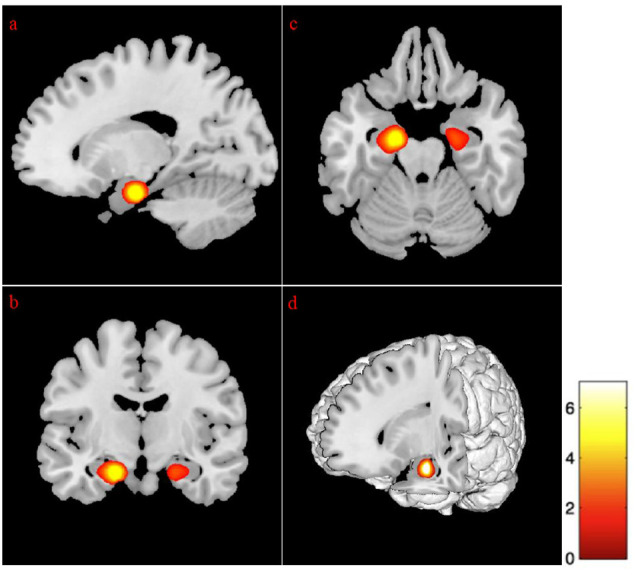
Regression between gray matter regions, depression, and advancing age. The position of the axes indicates a negative correlation between the right superior frontal gyrus medial segment (MNI coordinates: 6, 42, 21), advancing age, and depression. Color bar indicates the *t*-score for the regression slopes. **(A)** Sagittal plane, **(B)** coronal plane, **(C)** axial plane, **(D)** 3D illustration, *p* = 0.001.

Regression analysis revealed that advancing age and depressive symptomatologies were predictive of volumes in the left cingulate gyrus (anterior and middle) and the left subgenual anterior cingulate cortex. Additionally, the volume on the left lingual gyrus (*p* < 0.01), the left supramarginal, and the right precuneus gyrus (*p* < 0.01), was positively predicted by age and GDS scores. Also, the analysis revealed that the bilateral superior frontal gyrus (medial segment), the right dorsolateral prefrontal cortex, the right anterior prefrontal cortex, the right superior temporal gyrus, and the right hippocampus were negatively predicted by age and depression (Figure 3 and Table 7).

**Table 7:** Correlations between age, depression, and ROIs.

Structure	Side	MNI Coordinates			Peak-T	Age(↑) + GDS (↑)
		*x*	*y*	*z*		
Subgenual anterior cingulate cortex	L	−4	8	−10	6.30	−0.32*
Hippocampus	R	30	−18	−21	6.24	−0.32*
Anterior cingulate gyrus	R	4	26	26	5.97	−0.30*
Middle cingulate gyrus	R	4	21	28	6.03	−0.30*
Middle cingulate gyrus	L	−6	15	34	5.87	−0.29*
Supramarginal gyrus	L	−48	−28	15	5.76	−0.28*
Precuneus	R	2	−54	38	5.60	−0.27**
Superior temporal gyrus	R	44	2	−15	5.84	−0.29*
Primary auditory cortex	L	−40	−20	12	5.58	−0.27**
Anterior Prefrontal cortex	R	32	57	−4	5.41	−0.26*
Dorsolateral prefrontal cortex	R	46	42	4	5.75	−0.28*
Superior frontal gyrus medial segment	R	6	42	21	6.03	−0.30*
Superior frontal gyrus medial segment	L	−6	48	8	5.79	−0.28*
Lingual gyrus	L	−26	−44	−10	5.64	−0.27**

L, left; R, right; MNI, Montreal Neurological Institute; ↑ indicates an increase; *p < 0.01; **p < 0.001; *n* = 87.

## Discussion

NEUROAGE is the first longitudinal project on cognitive aging in Cyprus. It offers a unique opportunity to study the contribution of modifiable and unmodifiable risk factors to brain health. Study participants were community dwellers living independently, who were born and raised in a small Mediterranean island country with its unique geopolitical, social-cultural, and genetic characteristics. As noted in previous publications stemming from NEUROAGE (Philippou et al., 2018; Chadjikyprianou et al., 2021), the project has been able to capture the last generation of Cypriots who have attended very little formal schooling. Following the establishment of the Republic of Cyprus in 1960, public education has been free and mandatory through grade 9 or age 15. While the average education is 9 years, there was wide variability among study participants, ranging from 3 to 20 years.

Findings from the present study contribute to our understanding of the associations and predictive value of age, education, depression, and global and regional measures of brain volume on a cognitive screening measure in healthy aging. Age was inversely associated with global measures of brain volume, supporting arguments that as people age, gray matter (GM), and white matter (WM) volume decrease; whereas, cerebrospinal fluid (CSF) volume and volume-to-brain ratio (VBR) increase, with higher ratios of VBR indicating a greater loss in brain reserve (Carmichael et al., 2007).

Further evidence provided in the present study strengthens the aforementioned findings. Specifically, higher MMSE scores were associated with greater GM volume and higher levels of brain reserve. Furthermore, group comparisons revealed that individuals with higher MMSE scores presented with greater GM volume, compared to those with lower MMSE scores. While the MMSE group scoring 24–26 is still considered within normal cognition, without clinical findings of significant brain atrophy, the study captured significant differences in GM volume. Additionally, GM volume was predictive of cognitive performance, further supporting that GM volume can independently reflect the cognitive skills of elderly people. These findings extend existing literature supporting that healthy individuals with lower MMSE scores present with greater ventricular expansion (Ferrarini et al., 2008; Chou et al., 2009).

Following the associations between age and global GM volume, it was sought to further investigate specific regions of interest (ROIs) in healthy aging. Findings indicate that age was negatively associated with brain volume in the cortical-subcortical frontal, temporal, and fronto-thalamic regions and cerebellar areas. These include cortical structures such as the left side of the pars orbitalis, the medial frontal cortex, and the dorsal entorhinal cortex, the anterior cingulate, the right parahippocampal, the right superior temporal gyrus, the right basal forebrain, the right superior frontal gyrus, the left subgenual anterior cingulate cortex, and the left temporal pole; and subcortical structures such as the bilateral hippocampi, the right amygdala, the bilateral thalamus, and cerebellum exterior. Some of the above areas were also reported in a study by younger adults and future research may want to follow participants longitudinally or use large-scale predictive or machine learning paradigms to determine the patterns of atrophy across the age continuum (Bergfield et al., 2010).

However, none of the aforementioned areas associated with MMSE scores, which may underline that the atrophy in these regions may not be large enough, in healthy aging, to be detected *via* screening tools of global cognition; and that to detect such volume loss more comprehensive neuropsychological evaluation is required (Vyhnalek et al., 2014). It has been shown that lower MMSE scores clearly associate with decreased WM (e.g., splenium of corpus callosum) and GM (e.g., left temporal lobe), but only in people with AD (Apostolova et al., 2006; Baxter et al., 2006; Duan et al., 2006). In addition, the lack of associations between MMSE and ROIs in healthy aging may also be supportive of the previously stated argument that changes in brain volume precede any overt clinical symptoms. Longitudinal data will help delineate if these findings are suggestive of normal aging, or whether it is a prodromal sign of developing dementia at a later time.

Of particular interest was that participants with higher education (e.g., >12 years) presented with greater ventricular expansion, i.e., greater CSF volume, as well as lower brain reserve as compared to participants with fewer years of schooling (6–11 years). These findings provide support to the cognitive reserve (CR) hypotheses, a psychological construct measured through proxy indices. The CR hypothesis suggests that greater CR (i.e., the brain’s ability to apply cognitive strategies and compensate for already occurring pathological changes) can delay the onset of clinical symptoms even in the presence of dementia pathology (Scarmeas et al., 2006; Giogkaraki et al., 2013). While participants of the present study were healthy older adults who had been followed in our project for several years through our longitudinal cognitive testing, it is possible that some of them experienced pathological changes without overt clinical symptoms. Our most recent study analyzing our longitudinal data indicates that education affects cognitive performance, but does not affect the rate of change in measures of general cognitive screening like the MMSE across a 5-year period for cognitively healthy NEUROAGE participants (Chadjikyprianou et al., 2021). As the NEUROAGE database continues to grow, future studies should investigate the link between BR and CR in our longitudinal cohort. The present study revealed different patterns of volume loss between males and females (controlling for age, education, and depression) with male participants having greater total brain volume, GM, and larger ventricles. VBR was greater in males than in females, suggesting a greater degree of brain atrophy in male participants. It appears that the total brain volume difference between sexes is driven by the GM and CSF factors since there were not any significant WM differences between men and women. Previous research also demonstrated that males were at a greater risk of presenting with pathologically oversized ventricles than females (Missori et al., 2016). In the present study, males had more years of formal education than female participants, a key proxy measure of cognitive reserve (CR).

ROIs analyses also revealed different volume patterns across males and females. Female participants presented with less volume in their left temporal pole and left inferior temporal gyrus across the age continuum as compared to their male counterparts, an earlier onset of brain volume reduction in females. These findings support a region by sex by age dimorphism, also evident in the study of Taki et al. (2011).

A final contribution of the present study was the link between depressive symptoms, cognitive performance, and brain morphology. As expected, females presented with more depression symptoms than males. Also, participants with more depressive symptoms presented with less GM volume and underperformed on the MMSE. These findings suggest that screening for both depression and cognitive performance could aid in the early detection of clinical impairment, as both are related to reduced GM volume. Specifically, older adults with depressive symptoms exhibited less GM volume in the temporal and frontal lobes. Greater levels of depression in older adults were shown to predict less volume in the anterior (and middle) cingulate gyrus; a structure that relates directly with the control and regulation of mood. Present findings show that depression in healthy older adults could contribute to cognitive decline, highlighting the importance of early detection and treatment.

## Implications, Limitations, and Future Directions

The associations between age, education, brain volume, and cognitive performance presented in this exploratory study enhance our understanding of healthy cognitive aging. These findings support that global and regional brain changes may be on a continuum, in healthy aging and are influenced by age, education, and sex and by psychosocial factors such as depression. Of additional importance is the finding that the MMSE may be able to help in detecting changes in cognitive performance in specific cognitive domains such as orientation, attention, memory, language, and visual-construction skills, in individuals with reduced GM volume with no overt clinical signs; and that people who score between 24 and 26 and have lower education should be closely monitored for cognitive stability. However, it is worth mentioning that the MMSE is merely a screening tool and cannot provide an extensive assessment of the aforementioned areas. Furthermore, it does not assess all cognitive domains, such as executive functions and speed of processing, leaving these abilities untested. As demonstrated in this study, the MMSE is not adequate in detecting changes in ROIs and future research should incorporate extensive neurocognitive testing for exploring ROI associations and cognition in healthy aging. Finally, the addition of questions targeting historical and medical information should also be incorporated in the screening process to improve sensitivity (Demetriou and Constantinidou, 2018).

Education and depressive symptoms could moderate the brain changes and cognitive performance and future research should further explore these relationships and the role of both cognitive and brain reserve. Ideally, a longitudinal design could provide useful information on structural and cognitive changes that could denote transition to MCI and/or AD, along with biomarkers of AD (αβ42 and phosphorylated-Tau in CSF, etc.). Additional analyses incorporating T2-weighted images to study white matter hyperintensities could provide information on vascular dementia pathology in addition to volume atrophy associated more with AD pathology. As previously reported, in our NEUROAGE cohort, cognitive stability was demonstrated up to 5 years post baseline assessment on verbal episodic memory and executive functioning tasks in addition to gross cognitive measures like the MMSE (Chadjikyprianou et al., 2021). Finally, considering the exploratory nature of this study, the α-level remained at 0.05. Future studies could replicate these findings using a stricter α-level, thus controlling for multiple statistical tests.

## Data Availability Statement

The datasets presented in this article are not readily available because the dataset used in this study belongs to an ongoing longitudinal research project. Therefore, no data can be shared. Requests to access the datasets should be directed to fofic@ucy.ac.cy.

## Ethics Statement

The studies involving human participants were reviewed and approved by Cyprus National Bioethics Committee. The patients/participants provided their written informed consent to participate in this study.

## Author Contributions

EP conducted data analyses, wrote and revised the manuscript. EK conducted data analyses and wrote the manuscript. FC is the principal investigator of the study. She conceptualized and designed the project, coordinated the data collection, wrote and revised the manuscript. All authors contributed to the article and approved the submitted version.

## Conflict of Interest

The authors declare that the research was conducted in the absence of any commercial or financial relationships that could be construed as a potential conflict of interest.

## Publisher’s Note

All claims expressed in this article are solely those of the authors and do not necessarily represent those of their affiliated organizations, or those of the publisher, the editors and the reviewers. Any product that may be evaluated in this article, or claim that may be made by its manufacturer, is not guaranteed or endorsed by the publisher.
